# Supplementary material for: A KRAS-directed transcriptional silencing pathway that mediates the CpG island methylator phenotype
Source: eLife. 2014 Mar 12;3:e02313. doi: 10.7554/eLife.02313 (PMC3949416; doi:10.7554/eLife.02313)
Supplement: Supplementary file 1. — For shRNAs, clone IDs are provided, except in instances in which the shRNA clones have been discontinued, in which case sequences are provided. DOI: http://dx.doi.org/10.7554/eLife.02313.033 [file elife02313s001.docx]

**Supplementary File 1. List of shRNAs obtained from Open Biosystems/Thermo Scientific and synthesized siRNA sequences.** For shRNAs, clone IDs are provided, except in instances in which the shRNA clones have been discontinued, in which case sequences are provided.

| **Gene** | **Clone ID or Sequence (5’ 🡪 3’)** |
| --- | --- |
| *C2ORF82* (1) | TGCTGTTGACAGTGAGCGACCACCTCGGAAGCATTTAGAGTAGTGAAGCCACAGATGTACTCTAAATGCTTCCGAGGTGGGTGCCTACTGCCTCGGA |
| *C2ORF82* (2) | TGCTGTTGACAGTGAGCGAGGAAGCATTTAGAGATGTAAATAGTGAAGCCACAGATGTATTTACATCTCTAAATGCTTCCGTGCCTACTGCCTCGGA |
| *DICER1* (1) | TGCTGTTGACAGTGAGCGCCGAGTCTGGCTTTGAGAGTTATAGTGAAGCCACAGATGTAATATAATTAGAGATGGGTGCCCTGCCTACTGCCTCGGA |
| *DICER1* (2) | TGCTGTTGACAGTGAGCGAGGCACCCATCTCTAATTATATTAGTGAAGCCACAGATGTAATATAATTAGAGATGGGTGCCCTGCCTACTGCCTCGGA |
| *DNMT1* (1) | TGCTGTTGACAGTGAGCGCCGAGTCTGGCTTTGAGAGTTATAGTGAAGCCACAGATGTAATATAATTAGAGATGGGTGCCCTGCCTACTGCCTCGGA |
| *DNMT1* (2) | TGCTGTTGACAGTGAGCGCCGAGTCTGGCTTTGAGAGTTATAGTGAAGCCACAGATGTAATATAATTAGAGATGGGTGCCCTGCCTACTGCCTCGGA |
| *DNMT3A* (1) | V2LHS_74666 |
| *DNMT3A* (2) | V3LHS_391163 |
| *DNMT3B* (1) | TGCTGTTGACAGTGAGCGACCTGTTGTCATGAATGGCAAATAGTGAAGCCACAGATGTATTTGCCATTCATGACAACAGGGTGCCTACTGCCTCGGA |
| *DNMT3B* (2) | TGCTGTTGACAGTGAGCGAGGCCCATTTGACTTGTGTATTTAGTGAAGCCACAGATGTATTTGCCATTCATGACAACAGGGTGCCTACTGCCTCGGA |
| *F11R* (1) | TGCTGTTGACAGTGAGCGATCACCGCCTATCATCTGCATTTAGTGAAGCCACAGATGTAAATGCAGATGATAGGCGGTGAGTGCCTACTGCCTCGGA |
| *F11R* (2) | TGCTGTTGACAGTGAGCGAGAATTTACTTCTCTAGCTTACTAGTGAAGCCACAGATGTAGTAAGCTAGAGAAGTAAATTCCTGCCTACTGCCTCGGA |
| *KAP1* (1) | TGCTGTTGACAGTGAGCGAAGGACTACAACCTTATTGTTATAGTGAAGCCACAGATGTATAACAATAAGGTTGTAGTCCTCTGCCTACTGCCTCGGA |
| *KAP1* (2) | TGCTGTTGACAGTGAGCGAGCAATACAGACCATCAAGCAATAGTGAAGCCACAGATGTATTGCTTGATGGTCTGTATTGCCTGCCTACTGCCTCGGA |
| *KRAS* (1) | TGCTGTTGACAGTGAGCGAGGCTATATTTACATGCTACTATAGTGAAGCCACAGATGTATAGTAGCATGTAAATATAGCCCTGCCTACTGCCTCGGA |
| *KRAS* (2) | TGCTGTTGACAGTGAGCGCGCCTATGGTCCTAGTAGGAAATAGTGAAGCCACAGATGTATTTCCTACTAGGACCATAGGCATGCCTACTGCCTCGGA |
| *PRKD1* (1) | TGCTGTTGACAGTGAGCGCGGAAATTCCTTTATCTGAAATTAGTGAAGCCACAGATGTAATTTCAGATAAAGGAATTTCCTTGCCTACTGCCTCGGA |
| *PRKD1* (2) | TGCTGTTGACAGTGAGCGCCCAATTCACCTTGACAAGATTTAGTGAAGCCACAGATGTAAATCTTGTCAAGGTGAATTGGTTGCCTACTGCCTCGGA |
| *SETDB1* (1) | TGCTGTTGACAGTGAGCGCCCTGATAGTCAGCATGCGAATTAGTGAAGCCACAGATGTAATTCGCATGCTGACTATCAGGTTGCCTACTGCCTCGGA |
| *SETDB1* (2) | V3LHS_388251 |
| *SLC17A6* (1) | TGCTGTTGACAGTGAGCGATGCCACAGAGCTATATGTAATTAGTGAAGCCACAGATGTAATTACATATAGCTCTGTGGCACTGCCTACTGCCTCGGA |
| *SLC17A6* (2) | TGCTGTTGACAGTGAGCGCAGCAATAAGTGCAATGCATAATAGTGAAGCCACAGATGTATTATGCATTGCACTTATTGCTTTGCCTACTGCCTCGGA |
| *UBE2G2* (1) | TGCTGTTGACAGTGAGCGACCGACCATACTCATCAAAGATTAGTGAAGCCACAGATGTATTATGCATTGCACTTATTGCTTTGCCTACTGCCTCGGA |
| *UBE2G2* (2) | TGCTGTTGACAGTGAGCGAGGTATTATACCTATGTTGTATTAGTGAAGCCACAGATGTAATACAACATAGGTATAATACCGTGCCTACTGCCTCGGA |
| *USP28* (1) | TGCTGTTGACAGTGAGCGACCTGCATTCACCTTATCATTATAGTGAAGCCACAGATGTATTACCTGAAGAGGATACTGGCCTGCCTACTGCCTCGGA |
| *USP28* (2) | TGCTGTTGACAGTGAGCGAGCCAGTATCCTCTTCAGGTAATAGTGAAGCCACAGATGTATTACCTGAAGAGGATACTGGCCTGCCTACTGCCTCGGA |
| *ZNF304* (1) | TGCTGTTGACAGTGAGCGCCCTCTGTAGGGCAATGTGAAATAGTGAAGCCACAGATGTATTTCACATTGCCCTACAGAGGTTGCCTACTGCCTCGGA |
| *ZNF304* (2) | TGCTGTTGACAGTGAGCGAAAGGCCACTGTCAGAGGAAATTAGTGAAGCCACAGATGTAATTTCCTCTGACAGTGGCCTTGTGCCTACTGCCTCGGA |
| *ZNF304* (3) | V2LHS_58488 |
| **siRNAs** | |
| *CDX1* (1) | CAGTAAGCCTGTTGGATAAAG (Koslowski et al., 2009) |
| *CDX1* (2) | GGATGCAGCTTCAAGAATAAA (Koslowski et al., 2009) |
| *cJUN* (1) | GGCACAGCUUAAACAGAAATT |
| *cJUN* (2) | UUUCUGUUUAACGUGUGCCAC |

**References**

Koslowski M, Tureci O, Huber C, Sahin U. 2009. Selective activation of tumor growth-promoting Ca2+ channel MS4A12 in colon cancer by caudal type homeobox transcription factor CDX2. *Molecular Cancer* **8**: 77. doi:10.1186/1476-4598-8-77.
