# Supplementary material for: A KRAS-directed transcriptional silencing pathway that mediates the CpG island methylator phenotype
Source: eLife. 2014 Mar 12;3:e02313. doi: 10.7554/eLife.02313 (PMC3949416; doi:10.7554/eLife.02313)
Supplement: Supplementary file 2. — List of primers used for qRT-PCR, ChIP, PAT-ChIP and bisulfite sequencing. DOI: http://dx.doi.org/10.7554/eLife.02313.034 [file elife02313s002.docx]

**Supplementary File 2.** **List of primers used for qRT-PCR, ChIP, PAT-ChIP and bisulfite sequencing.**

| **Gene** | **Forward primer (5’ 🡪 3’)** | **Reverse primer (5’ 🡪 3’)** |
| --- | --- | --- |
| **qRT-PCR** | | |
| *p14^ARF^* | CCCTCGTGCTGATGCTACTG | ACCTGGTCTTCTAGGAAGCGG |
| *P15^INK4B^* | GGCGCGGGGACTAGTGGAGA | GCCCATCATCATGACCTGGATCGC |
| *P16^INK4A^* | GAAGGTCCCTCAGACATCCCC | CCCTGTAGGACCTTCGGTGAC |
| *ABTB2* | GCTGGTCAGTTTGTTGCTGA | TGGCTGAAGCAGTTCATGTC |
| *ADAMTS1* | GGATGGCTGATGTTGGAACT | TAATTCATGGGCTGTGGTGA |
| *ALX4* | AGAGAGCAACAAGGGCAAGA | CACGTCTGGGTAGTGGGTCT |
| *AOX1* | TCACTCACGGTGGAATTGAA | CAGGTGGACATTCGACATTG |
| *BLAST^R^* | GTCGCCAGCGCAGCTCTCTC | AGTCAGGTTGCCAGCTGCCG |
| *BNIP3* | TTCCTTCCATCTCTGCTGCT | ATCAAAAGGTGCTGGTGGAG |
| *C2orf82* | GGGCTCCTGTGGAACATC | TTAACCAGCGCAGTCCTCC |
| *CACNA1G* | AAGCAGACAGTGGAGCCTGT | TCTGAGTCAGGCATTTCACG |
| *CDO1* | GTACGCCAAGTTCGACCAGT | GTCCTTCACCCCAACAGAGA |
| *CDX1* | CACCTATCCACCCTCTGCAT | GACTCCCTTATCCCCCAGAG |
| *CHFR* | CCTCTGTGGCAAGTGATGAA | TCCAAATCCTCCTGATCCTG |
| *CIDEB* | GGAACTGCAGTGGACAGTGA | CACTCCTTGTAGGGCTCCAG |
| *cJUN* | GGTAGCAGATAAGTGTTGAG | GGCGCTAGCTCTGGGCAGTT |
| *CRABP1* | GCAAGTGCAGGAGTTTAGCC | CACGGGTCCAGTAGGTTTTG |
| *CLDN23* | TTGCCATGCAAACTCTCAAG | CCATTAAGCTGCTGGCATTT |
| *COL4A2* | AAGGAATCATGGGCTTTCCT | CTCTGGCACCTTTTGCTAGG |
| *DFNA5* | GAATGAGGTCCTGTGCGTTT | GATGCCACCACACTTCTCCT |
| *DICER1* | GTACGACTACCACAAGTACTTC | ATAGTACACCTGCCAGACTGT |
| *DNMT1* | TCCACAGCAAAGTGAAAGTCATCT | CTCGCGTAGTCTTGATCATACCA |
| *DNMT3A* | CACACCTGAGCGCGACTGCA | ACGATCCACGCGCCCATTCC |
| *DNMT3B* | CCCCGGAGATCAGAGGCCGAA | CCCGCCGTCTCAGGGACTGT |
| *EDIL3* | TTGGCTGATGGTTCCTTTTC | ATGGCATGGATTAGGAGTGC |
| *EFEMP1* | CAGGGACGCACAACTGTAGA | ATTGAAACCCAGGACTGCAC |
| *EFHB* | CCTCCCTAATGTCCCAGGAT | CCTCAGACCAGAAGGCTGAC |
| *EFHD1* | GATGGCTTCATCGACCTGAT | GTCCTCATCCACCTCCTTGA |
| *ELMO1* | CTGCTCAGCATGGAAATCAA | TCATAGTTGCTGGGCTCCTT |
| *F11R* | CTATAGCCGAGGCCACTTTG | ACACCAGGAATGACGAGGTC |
| *FBN2* | TCCTGGATATCAGGCTACGC | TGAATTTGTGCACTGGGTGT |
| *HAND1* | GTCCGCAGAAGGGTTAAACA | GGCAAGGCTGAAAATGAGAC |
| *ID4* | GGGTGGGCTACTTTTCTTCC | GTCGCTCTGGGTTTTACGAG |
| *IGFBP3* | AGGGCACTCTGGGAACCTAT | TGCAGTCATCCGAAGAATTG |
| *IGFBP7* | GGCATGGAGTGCGTGAAGAG | CTTGCTGACCTGGGTGATGG |
| *IRF8* | AGTGGCTGATCGAGCAGATT | AGTGGCTGGTTCAGCTTTGT |
| *KAP1* | AATGATGCCCAGAAGGTGAC | TTGAGGTCCCACTGAAACTT |
| *KRAS* | TAGACACAAAACAGGCTCAGG | TAATTACACACTTTGTCTTTGA |
| *LOX* | ATATTCCTGGGAATGGCACA | CCAGGACTCAATCCCTGTGT |
| *LRP2* | AAACAATGGTGGGTGCTCTC | TTCTTGCCATCACTTTGCAG |
| *NDNF* | CGCTCCCTGCAGTTTAAAAG | AAGTTGCTGCGAAGTGGAGT |
| *NEUROG1* | GTTACTTTCCCCCTCCCCTA | CTTTAAAGCTCCCGCTTCCT |
| *OVOL1* | CAGGGCTTCTAATGCTCAGG | AGTGCACACACACAAGCACA |
| *PENK* | AAGCCAAAGAGCTGCAGAAG | TTCAGGAAACCTCCATACCG |
| *PPP1R14A* | CTGGACGTGGAGAAGTGGAT | AGCAGCTCCTGGATGAAGTC |
| *PPP1R3C* | TTGCAAGAGCGAACAGTGAC | TGCTCAGTTGGAATGACAGG |
| *PRKD1* | GGGGCTTTTCAGGCAGGGCT | ACTCTGCCCCAGGGCTAAGCA |
| *RASSF2* | GGTCTTCCTGCACTTGAAGC | GCATCTCCACACACAAGGTG |
| *SEPT9* | CATCACGCACGATATTGAGG | CCAGCAGTTCTCGTTGTTGA |
| *SETDB1* | ACATCCTCAGCCTCTGCAT | TTCCAGTACCGGTCAGATCC |
| *SFRP1* | AAGGGAGGCTCTCTGTAGGC | AATGACCAGGCCAATCAGTC |
| *SFRP2* | GGGTCTGGTTGGTTGTTGTT | GGGCCACAGAGAAAATTGAA |
| *SLC17A6* | TCGGCCAGATCTACAGGGTGCT | CACAGCGGCGCCTTCCTCTC |
| *SLC30A10* | ATCCACAATGTGACCATCCA | CTTGGAGATGCAGGGTGAGT |
| *SOCS1* | CTCCTTCCCCTTCCAGATTT | CACATGGTTCCAGGCAAGTA |
| *SPON1* | CACATTTGATGGGGTGACTG | TGTCTTCTCGGACCAATTCC |
| *STOX2* | GGCGAACTCAACTCTTGTCC | TTCTTTCCCAGAGGTGATGG |
| *THBD* | CGGGTTGTGTGTCTGTTCAC | CCTCCATGCATCTCATAGCA |
| *THBS1* | ACCAAAGCCTGCAAGAAAGA | TCTGTACCCCTCCTCCACAG |
| *THBS2* | AAGTGTGTGAGCCCGAAAAC | GTACATGGGGTCGCTGAAGT |
| *TMEFF2* | CAATGGGGAGAGCTACCAGA | TCTGTGGCACATGATCCTTC |
| *TOLLIP* | CGGTGGTACAGAGAGCCTTC | ACCACTTGTCCTCCACCTTG |
| *TP73-AS1* | GGGTAACTCCCCACTGTTGA | GGCTGAGCTGGACAAAAGAC |
| *TSPYL5* | TCCCACTTTCAGCAGTCCTT | CCCAGGAGAAGCTTGAGATG |
| *UBE2G2* | CAGCTGTTGCGGGGCCATGG | GGGCCTGCTACAATTCCTTCCGGA |
| *UCHL1* | AGCGTGAGCAAGGAGAAGTC | TTGAAGGGAAGAGGGGAAAT |
| *USP28* | AGGGGCCATGGTGGAGGGTG | TCTGGCTGCCCAAGGGACTGA |
| *VIM* | GGCCCAGCTGTAAGTTGGTA | CCTAGCGGTTTAGGGGAAAC |
| *ZNF304* | AAGAGCTGTACAGTCCACATG | TCTCACCTCAGCATGAGTC |
| *ZSCAN18* | GCTTTCCTGCAGCCATTTAG | TTGAAGAAAGCACTGGCAGA |
| **ChIP** | | |
| *p14^ARF^* | GTGGGTCCCAGTCTGCAGTTA | CCTTTGGCACCAGAGGTGAG |
| *P15^INK4B^* | GGAACCTAGATCGCCGATGTAG | TGTTTTACGCGTGGAATGCAC |
| *P16^INK4A^* | ACCCCGATTCAATTTGGCAG | AAAAAGAAATCCGCCCCCG |
| *AOX1* | ATCCTGGCTGTGGGTAACTG | TATCGCTAGCGCATTCTCCT |
| *CACNA1G* | GTCTGGGCAGCAGTCTGATT | GGAGAGAACCACAGCTGGAA |
| *CHFR* | TGTGCAACTGTACCCGAAAG | ATTCTGAGAGCCCCGCTAAT |
| *EFEMP1* | TCCACCAACAGCATACAAGC | TGTGAGGTGGGGTTTGTTTT |
| *GCLC* | ACCGCCTCCCCGTGACTCAG | CAGCAGCAGCAGCCCAGAGG |
| *HAND1* | GATAGCCACTCCCCCTTTTC | CGGCTTTGATGTCAACCTCT |
| *IRF8* | AATATCCAGCGCTCGTGAAG | GGCCCATTAATCAGAATCCA |
| *LOX* | GCCAGAATAAGACCGTGAGC | AGAACCCCAATCCCAGAGTT |
| *PRKD1* | GCTCGGCTAAATTTTGCATT | GGGTGACAGAGCAAGACTCC |
| *USP28* | GCGTGGTGACTCATGTCTGT | CCCAGGATGGTCTCAAACTT |
| **PAT-ChIP** | | |
| *p14^ARF^* | GTGGGTCCCAGTCTGCAGTTA | CCTTTGGCACCAGAGGTGAG |
|  |  |  |
| **Bisulfite sequencing** | | |
| *p14^ARF^* | TGGAAAGGAAGAAGGGAGAGAGTTTAG | ACCACCATCTTCCCACCCTC |
| *p14^ARF^* (nested) | GATGTGGAAGAAAAGGGGAGGA | TCCCCTCATCAAAAATCTTCCA |
| *p14^ARF^-reporter* | TGGAAAGGAAGAAGGGAGAGAGTTTAG | AATCACCAATAAAACCTAAAACCCAAAACAACAA |
| *p14^ARF^-*reporter (nested) | GATGTGGAAGAAAAGGGGAGGA | CCCATAACAAAAATATATCTATCTCCATAATAT |
| *AOX1* | ADS2444* | |
| *CACNA1G* | ADS2300* | |
| *CHFR* | ADS1254* | |
| *EFEMP1* | ADS1462* | |
| *HAND1* | ADS043* | |
| *IRF8* | ADS1690* | |
| *LOX* | ADS852* | |
| *P14^ARF^* | ADS2130* | |
| *P16^INK4A^* | ADS1067* | |

*Commercially available from EpigenDX.
